# Solitary Amyloid Tumor of the Tongue Base

**DOI:** 10.1155/2009/515068

**Published:** 2009-01-06

**Authors:** S. Akyildiz, B. Doganavsargil, S. Göde, A. Veral

**Affiliations:** ^1^Otolaryngology Department, Ege University Medical Faculty Hospital, Bornova, 35040 Izmir, Turkey; ^2^Pathology Department, Ege University Medical Faculty Hospital, Bornova, 35040 Izmir, Turkey

## Abstract

The purpose of this article is to present a rare case of localized, solitary amyloid tumor of tongue base and emphasize some of the characteristic features of challenging clinical and histopathologic diagnosis. In this paper, we focused on the clinical and pathological specifications of this rare tumor, so any unnecessary examinations or measures may be spared. Negative staining of amyloid material with AAC and osseous metaplasia noted in the histopathologic examination may not be thought as definite criteria for localized amyloidosis, but a supporter of localized, solitary amyloid tumor diagnosis.

## 1. Introduction

Amyloid can affect any site in the head and neck including the orbit, sinuses, oral cavity, salivary glands, pharynx, and larynx. Amyloid involvement of tongue is almost always secondary to systemic amyloidosis and localized involvement is extremely rare [1, 2].

The etiology, treatment, and outcome of systemic amyloidosis are totally different from localized amyloidosis. The mean survival of patients with systemic amyloidosis is between 5 to 15 months, whereas patients with localized amyloidosis have excellent prognosis [1].

To rule out a systemic amyloidosis for these patients is extremely critical because this can markedly change the expected morbidity and mortality [1]. Abdominal fat aspirate is a risk-free procedure and is the most specific test available as it is positive in 70–80% of patients with amyloidosis; however, the sensitivity is low and is very technique sensitive [2, 3].

The purpose of this article is to present a rare case of localized, solitary amyloid tumor of tongue base and emphasize some of the characteristic features of challenging histopathologic diagnosis.

## 2. Case Report

A 61-year-old male patient presented with pyrosis of tongue and a nodular mass in tongue base. Head and neck examination revealed a 1 × 1 cm well-circumscribed, rubbery, nodular mass at the right side of the tongue base. His review of systems was negative for symptoms including fever and weight loss.

**Figure 1:**
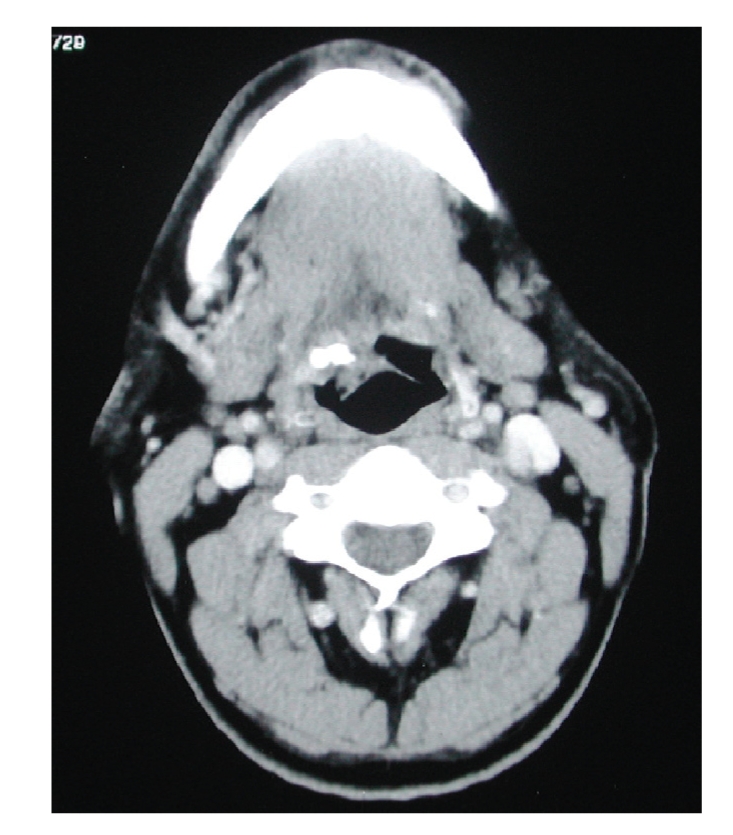
CT scan of the lesion. The lesion can be seen on the right side of tongue base.

A computed tomography was performed and a possible granulomatous or inflammatory mass with calcification was reported (Figure 1). An incisional biopsy was performed from the mass preoperatively. Histologic examination revealed a benign lesion without forming a significant mass. The lesion appeared to be under normal mucosa, within hyalinized muscular and connective tissue which includes focal chondroid differentiation areas. Transcervical total excision of the lesion was planned.

**Figure 2:**
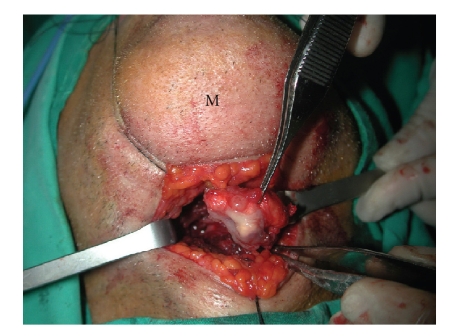
En bloc excision of well-circumscribed mass in the tongue base. M: mentum.

Rough and irregular mass, 2 cm in diameter, was dissected from surrounding normal tissue with minimal tissue loss and en bloc excision could be achieved via transcervical suprahyoid approach (Figure 2).

**Figure 3:**
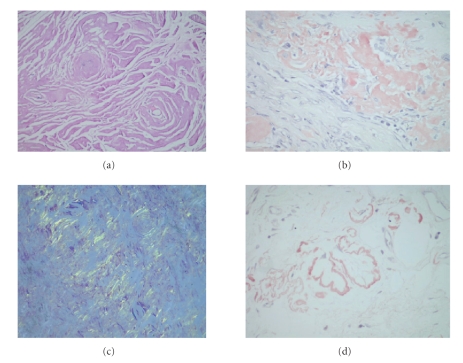
(a) Closer view of amorphous, fibrillary matrix deposition (hematoxylin and eosin, ×20). (b) Peach-red staining of the material in Congo-red staining (Congo-red, ×20). (c) Apple-green birefringence under polarized light (Congo-red, polarization microscope, ×20). (d) Amyloid deposition in vessel walls (Congo-red, ×20).

**Figure 4:**
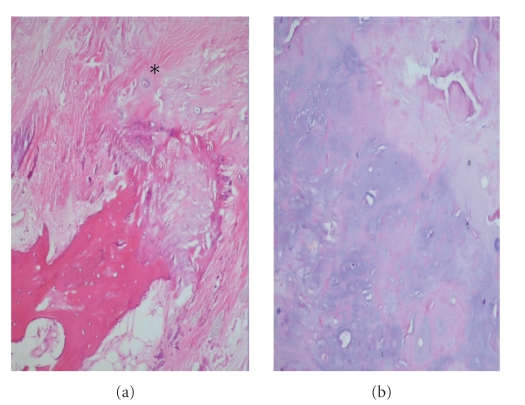
(a) Osseous metaplasia observed adjacent to amyloid deposition. Note the single chondrocytes spread in amyloid matrix (asterix) (hematoxlin eosin, ×10). (b) Areas presenting extensive chondroid metaplasia (hematoxlin eosin, ×10).

Postoperative microscopic examination of the specimen revealed a well-circumscribed submucosal lesion characterized with structureless eosinophilic material deposition. Overlying mucosa was inflamed and focally eroded. The amorphous, more or less homogenous eosinophilic material resembled amyloid histologically and on Congo red staining, exhibited the classical apple-green birefringence under polarized light. Deposits of amyloid were also found in and around the walls of small blood vessels. Immunohistochemical examination of the matrix with monoclonal antibody AA-Congo (AAC) (Dako, Denmark 1/100 dilution) revealed a non-AA-type amyloid deposition (Figure 3). At the periphery of the lesion, there was a prominent chronic inflammatory reaction rich in mixed kappa and lambda immunoreactive (Neomarkers, Barcelona, Spain 1/3000, 1/100 dilutions) plasma cells. A giant cell response to amyloid material was also present. Interestingly, there was a metaplastic bone tissue surrounding the mass. Focal chondroid differentiation was also observed either neighboring the osseous tissue or spread as single chondrocytes in the amyloid matrix (Figure 4). Thus, the lesion was reported as localized amyloid tumor of tongue base with focal osseous and chondroid metaplasia.

Diagnostic workup including complete blood count, liver and renal function tests, urine analysis, esophagography, chest X-ray, electrocardiography, echocardiography, bone-marrow biopsy, ESR, RF, ANA, and abdominal fat biopsy was carried out.

All examinations for exclusion of systemic amyloidosis, including bone-marrow and abdominal fat biopsies, were found to be negative for Congo red staining and amyloid deposition. Bone-marrow aspiration biopsy was found to be free of any type of infiltrations. Thus, the patient was determined to be free of systemic amyloidosis and diagnosed as localized, solitary amyloid tumor of the tongue base.

Annual examinations were performed in the four-year follow-up period.

## 3. Comment

The differential diagnosis of the mass in tongue base includes neoplastic processes and also lingual thyroid. Because the mass was settled under normal appearing mucosa, a preoperative incisional biopsy was performed in order to rule out thyroid tissue and malignancy. We could not achieve a definitive pathology with preoperative biopsy so we planned total excision of the mass.

When amyloidosis is the histopathologic diagnosis in the patients presenting with a localized mass in head and neck region, the main diagnostic dilemma of the surgeon becomes the extension of the disease. Many diagnostic examinations were proposed to rule out a systemic disease. Histopathologic examinations of these lesions may help clinicians about the necessity of the diagnostic examinations. Amyloid tumor of tongue base is a rare condition and may not be predicted preoperatively, therefore there is little information on the clinical and histopathologic features of the disease. In this paper, we focused on the clinical and pathological specifications of this rare tumor, so any unnecessary examinations or measures may be spared and patient may be informed clearly about the disease.

Also, in the literature there is no consensus on terminology of the disease. We did not prefer to use the term “localized amyloidosis of tongue base” for this disease because diffuse involvement of tongue with systemic amyloidosis may be mistaken with well-circumscribed amyloid tumor. The terms “localized primary amyloid tumor” and “solitary amyloid tumor” were both used for localized amyloid deposits without systemic amyloidosis or multiple myeloma in the literature, and better distinguish from the systemic form and better defines the disease. So we decided to use the term “solitary amyloid tumor” for the diagnosis after ruling out systemic involvement of the disease because it briefly underlines the nature of the disease.

The definitive treatment of localized amyloidosis was cited to be surgery. Surgery alone may be 100% curative [1]. The diagnostic workup of our patient was performed after histopathological diagnosis. Patient's urine immunofixation was negative for Bence Jones protein. Bone-marrow examination revealed no evidence of a plasma cell dyscrasia. Also, abdominal fat biopsy was free of amyloid deposits. To date, the patient has not developed clinical or laboratory evidence of systemic amyloidosis or multiple myeloma for a four-year follow-up period.

Although, negative staining with AAC and osseous metaplasia which is noted in histopathological examination supports non-AA-type amyloidosis, we performed these examinations to support our diagnosis. Negative staining of amyloid material with AAC is mostly seen in primary type of amyloidosis. Also, osseous metaplasia noted in the histopathologic examination may not be thought as definite criteria for localized amyloidosis, but a supporter of localized, solitary amyloid tumor diagnosis. There is data about osseous metaplasia in the literature regarding the solitary amyloid tumor of other tissues [4], and to our knowledge this is the first case in tongue. This osseous metaplasia correlates with the calcification on computed tomography.

A submucosal, surgically well-defined, well-circumscribed, and rubbery mass in tongue base with calcifications on radiologic studies may not be surgically differentiated from other tumors of tongue. When histopathology reveals amyloid deposits on light microscopy, these clinical findings may come out as important features in order to differentiate local disease from involvement of tongue by a systemic disease.

With this little information regarding the clinical and histopathologic features of localized, solitary amyloid tumor of tongue base, we may not discuss the necessity of the examinations in order to rule out systemic disease including abdominal fat and bone-marrow biopsy. Therefore, the publication of more cases or series of the disease may yield better characterization of the histopathologic, radiologic, and clinical features of solitary amyloid tumor of tongue base.
